# Case report: Rare case of multinodular and vacuolar neuronal tumors in the cerebellum

**DOI:** 10.3389/fneur.2023.1309209

**Published:** 2024-01-05

**Authors:** Zhou Wang, Jiwei Ma

**Affiliations:** Department of Pathology, Shandong Provincial Hospital Affiliated to Shandong First Medical University, Jinan, Shandong, China

**Keywords:** multinodular and vacuolar neuronal tumors, cerebellum, case report, literature review, neuro-oncology

## Abstract

Multinodular and vacuolar neuronal tumor (MVNT) is a rare and benign neuroepithelial tumor. Most reports describe tumors located in the cerebral hemisphere. A literature review found that 15 cases were located in the posterior cranial fossa, but all lacked pathological evidence. In this case, a patient sought medical attention due to insomnia and irritability. Neuroepithelial tumors were found in the imaging, and the patient underwent radiation therapy. Three years later, malignant tumors were found upon imaging examination. After surgical resection and pathological testing, MVNT occurring in the cerebellum was diagnosed. MVNT is rare in the cerebellum, and direct imaging diagnosis becomes difficult after treatment. Therefore, our report of this case helps to further accurate understanding of the imaging, pathological, and molecular genetic changes occurring before and after MVNT treatment, and will improve the accuracy of pre-treatment diagnosis and reduce the likelihood of overtreatment.

## Introduction

Multinodular and vacuolating neuronal tumor (MVNT) was first reported in 2013 (1). MVNT was classified by the World Health Organization Classification (WHO) under Tumors of the Central Nervous System in 2016, and then as a distinct WHO grade 1 tumor type in the 2021 WHO classification (2). Histologically, MVNTs are combined with well-defined and coalescing cytoplasm and matrix nodules and are located mainly in the cerebrum. To date, 15 cases have been described to involve the posterior fossa, as diagnosed by radiography, but have not been verified by pathology. This report describes the case of a 53-year-old woman with an MVNT located in the right cerebellar hemisphere, verified by histology, immunophenotype, and molecular pathology.

## Case report

**Figure 1:**
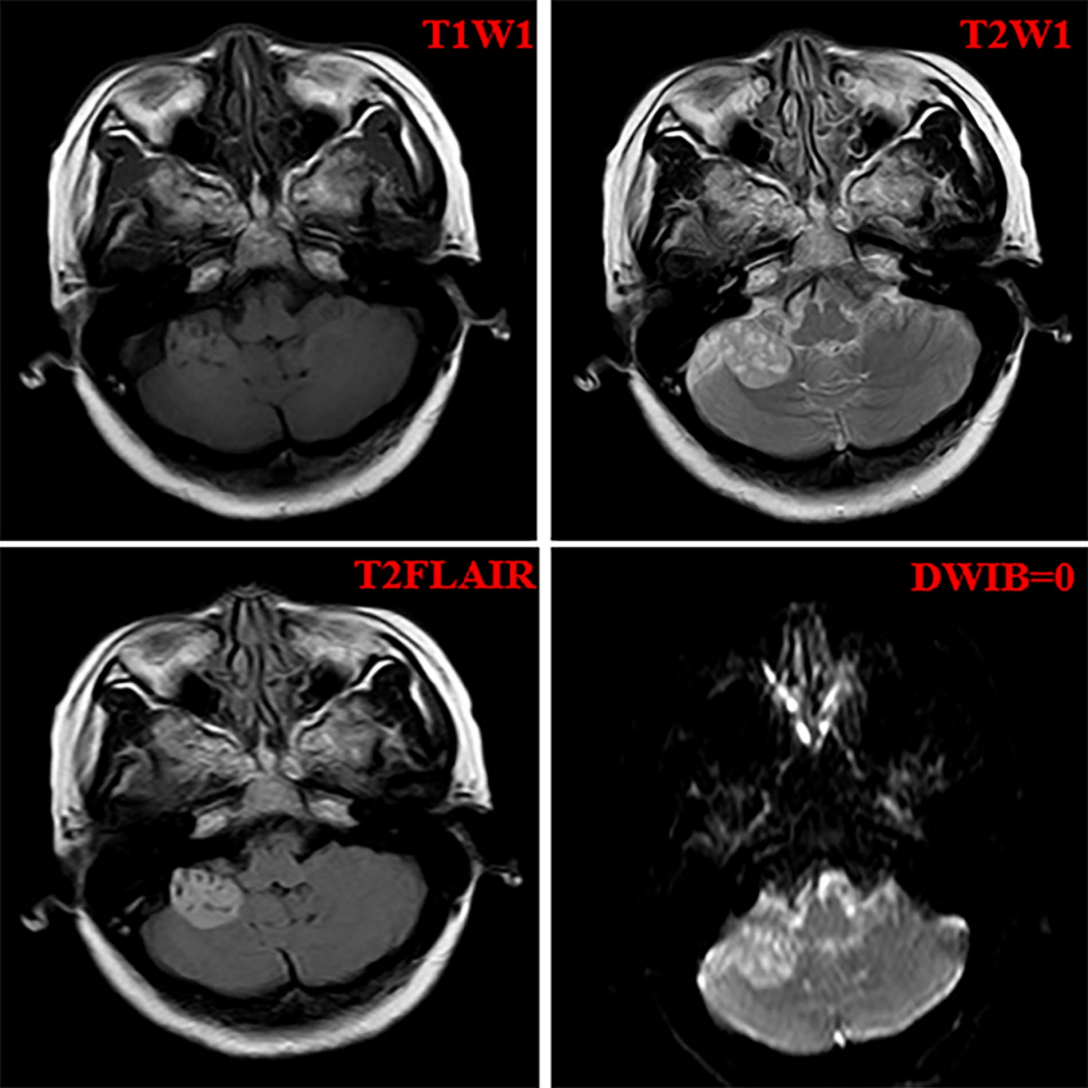
The imaging features of the local hospital including T1WI, T2WI, T2Flair, and DWIB images.

**Figure 2:**
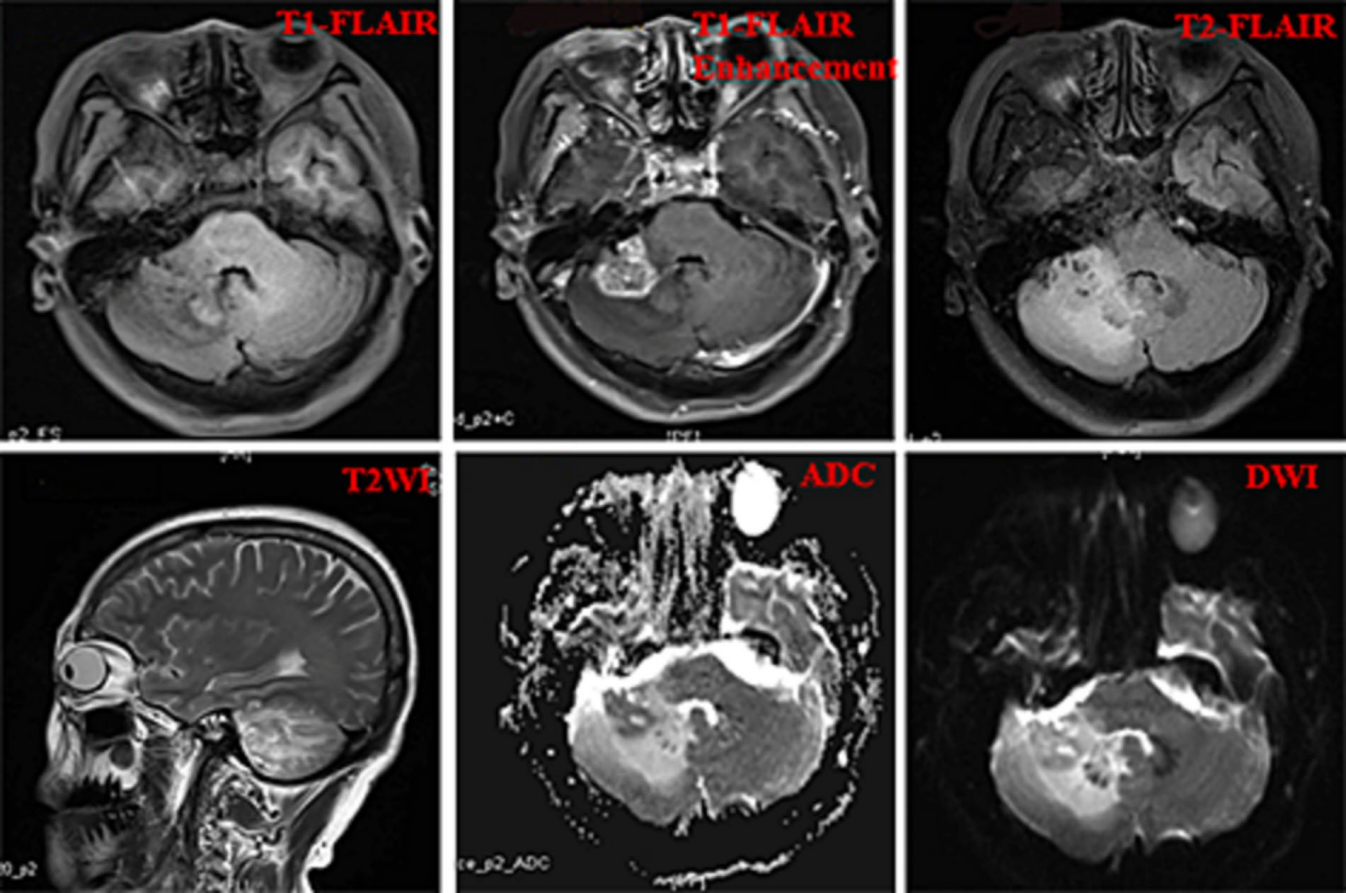
The imaging results of our hospital including T1Flair, T1Flair enhancement, T2Flair, T2WI, ADC, and DWI images.

**Figure 3:**
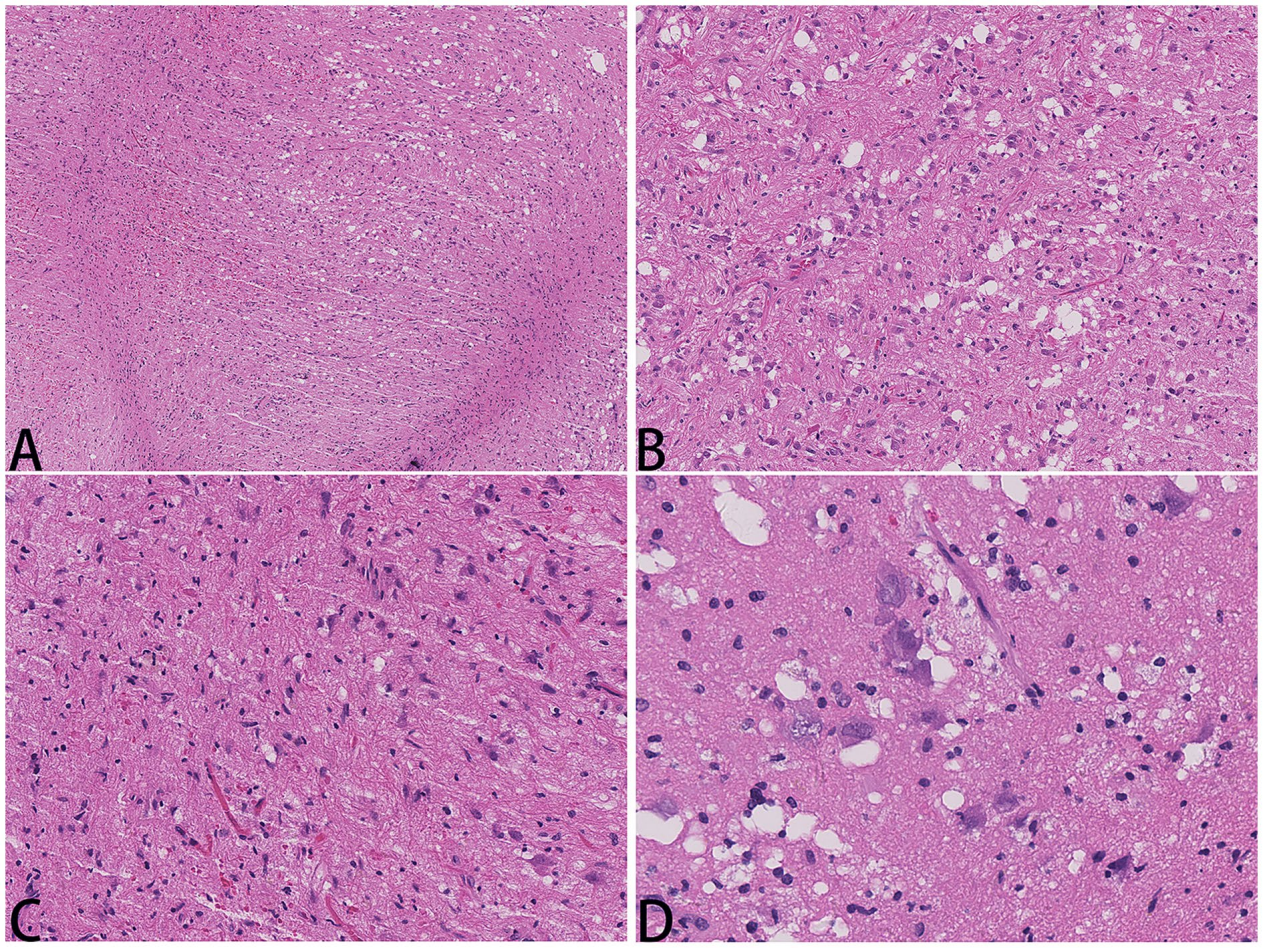
Hematology and eosin staining images of MVNT; The magnification in **(A)** is 40x, **(B, C)** are 200x, and **(D)** is 400x.

**Figure 4:**
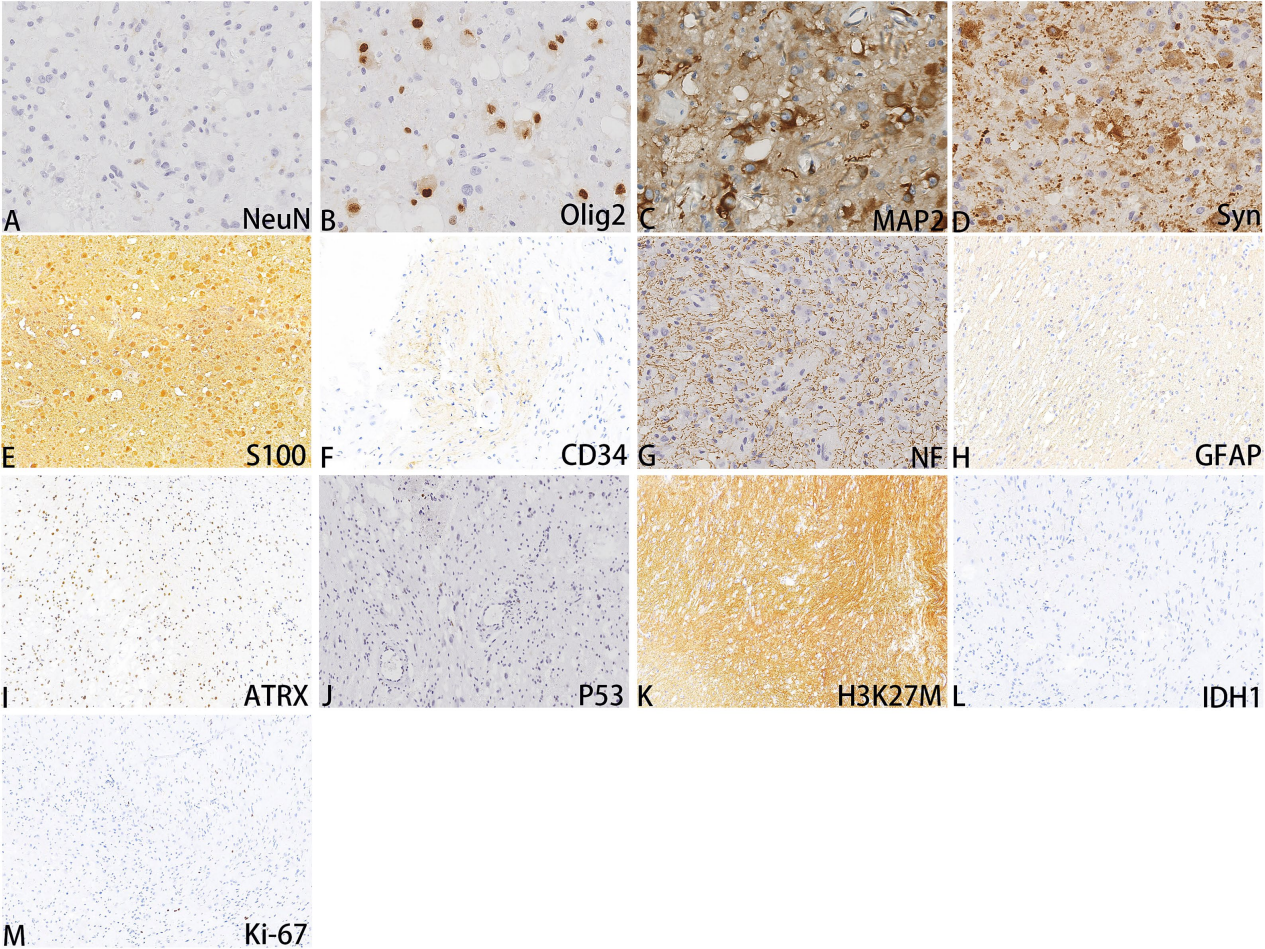
Immunohistochemistry images of MVNT **(A-M)**; The magnifications are 400x.

A 52-year-old woman sought medical attention at a local hospital three years ago due to insomnia and irritability. Magnetic resonance imaging (MRI) revealed neuroepithelial tumors in the cerebellum. Radiotherapy was administered three times for 40 min each time, followed by a follow-up examination every six months. The patient experienced dizziness, with unstable walking for two months prior, and sought neurosurgical treatment. The neurological examination and laboratory results were normal. Previous physical health and family and genetic history were unremarkable. Local hospital MRI revealed a quasi-circular mass on the convex surface of the right cerebellar hemisphere, with a size of approximately 2.5 cm × 2.0 cm, and with slightly shorter T1 and longer T2 signals visible. T2 FLAIR showed mixed high and low signals, with slight enhancement in enhanced scans. A few patchy areas of slightly longer T1 and T2 signals could be seen in the white matter areas of both cerebral hemispheres. T2 FLAIR showed high signal intensity, while DWI showed the same signal intensity. A low-grade glioma, excluding neurogenic tumors, was considered (Figure 1). Our hospital’s MRI showed irregular equal length mixed signal lesions of T1 and T2 in the right cerebellar hemisphere, and T2 FLAIR showed mixed high and low signals. DWI and ADC images showed uneven diffusion limitation, with a maximum cross-section of about 3.0 cm × 2.8 cm. A patchy edema zone surrounded the lesion, and the enhanced scan showed uneven enhancement. Multiple abnormal nodular enhancement lesions were seen in the cerebellar sulcus and the base of the adjacent occipital lobe around the lesion, with a few punctate T1 long T2 signals in the bilateral frontal cortex. T2-FLAIR showed a high signal, and DWI did not show an abnormal diffusion restriction (Figure 2). A malignant tumor was considered based on the MRI results of our hospital. Due to imaging considerations for malignant tumors and signs of unstable walking, neurosurgeons performed cerebellar tumor resection surgery for 2 h on the patient. The surgical process was smooth. Pathological examination of the excised tumor revealed that the lesions were distributed in nodular shapes of varying sizes, with independent nodules separated by glial tissue (Figures 3A,C). Tumor cells within the nodules were larger, with scattered neuronal-like cells. Neuron-like nuclei were ovoid, nucleoli were prominent, the cytoplasm was eosinophilic or vacuolate, and eccentric vacuoles of varying sizes surrounded the cells. Occasional binuclear cells were observed. There were no obvious cellular atypia or pathological mitosis. (Figures 3B,D). Immunohistochemistry revealed negative NeuN staining and positive olig2 staining in neuron-like cells (Figures 4A,B). Neuron-like cells also showed positivity for MAP2, Syn, S-100 (Figures 4C–E). Immunohistochemistry also showed positive NF staining of process (axons) in the background of neuron-like cells. Positive staining of GFAP was found in astrocytes, which were surrounded with neuron-like cells. CD34 positive staining of multipolar cells was found in the focal cortex away from the nodules area (Figures 4F–H). There was no evidence of deletion in ATRX, no sense mutations in P53, or mutations in H3K27M or IDH1, and approximately 2% positive expression of Ki-67 (Figures 4I–M). MVNT is associated with small indel or hotspot mutations in the MAP1K2 or MAP2K1 genes related to the MAPK pathway. A non-frameshift deletion mutation in the second exon of MAP2K1 was identified by high-throughput sequencing: MAP2K1 c.165_179 del (p.Q56_V60del). This mutation can lead to the activation of the MAPK pathway (3).

Based on comprehensive imaging, histological morphology, immunohistochemistry, and molecular pathological examination, this case is diagnosed as a WHO grade 1 MVNT occurring in the cerebellum.

Patient compliance was good, and there were no diagnostic challenges. The patient did not experience any significant discomfort during hospitalization and improved walking instability 6 days after surgery. As MVNT is a benign tumor, no other adjuvant treatment was performed after resection, so only follow-up observation was conducted. After 12 months of follow-up, computed tomography (CT) scans showed no progression or recurrence, and the patient reported no physical discomfort.

## Discussion

MVNT was first described by Huse et al. in 10 similar cases (1). As of June 2023, we reviewed and analyzed 112 reported cases of MVNT, ranging in age from 5 to 77 years. There were 60 cases in women (53.57%), 48 cases in men (42.85%), and 4 cases without mention of sex. The diameter of the tumor ranges from 1 mm to 78 mm. The main site of tumor onset is concentrated in the cerebral hemisphere (frontal, occipital, and temporal lobes), with 15 cases reported to be located in the cerebellar hemisphere that were not verified by pathology. The patient’s symptoms were mostly headache and seizures. In terms of treatment, 42 patients underwent surgical resection. Due to MVNT being considered a “leave me alone” lesion (3), 41 cases were chosen that were not treated, with follow-up observation. 40 cases underwent follow-up observation from 5 months to 144 months, and no recurrence or progression was observed (Supplementary Table S1).

In the reported cases, MRI showed hypointense or isointense signals on T1W, hyperintensity on T2W, and FLAIR weighted with a multinodular appearance (Supplementary Table S2). Pathological examination of the samples selected for surgical resection revealed multiple nodular areas within the white matter, where neuron-like cells were visible. The nucleus of neuron-like cells was circular and ovoid, with an eosinophilic cytoplasm and vacuoles visible both inside and outside the cytoplasm. Cells were mildly atypical, and nodes were separated by glial tissue. Neuron-like cells HuC/HuD, MAP2, SYN, and Olig2 were positive in immunophenotype; NeuN, CgA, and IDH1 were negative; GFAP, and CD34 between nodules were positive. Fifteen patients underwent genetic testing, and 4 showed mutations in the MAP1K1 or MAP2K1 genes, including in-frame deletion in exon 2 of MAPK2K1 of MAPK pathway results in the p.Q56_V60del mutation, MAP2K1 P.K57-E62delinsK, MAP1K1 p.Q56P (c.167A > C), and MAP2K1 P.Q56P (c.167A > C). Two cases of BFAF gene mutations included BRAF (I597R and G469S), and one case harbored an FGFR-INA gene fusion (Supplementary Table S3).

Currently, 15 cases have reported lesions in the cerebellum but were not confirmed by pathology (4–7). The case described in this report is the only MVNT confirmed by pathology and molecular testing results in the infratentorial cerebellum. Unlike MVNT patients who experience headaches or epilepsy as their initial symptoms in the cerebral hemisphere, this patient presented initially with dizziness and unstable walking. The typical CT/MRI manifestation of MVNT is nodular lesions along the contour of the gyrus, involving deep cortical layers and adjacent white matter. In addition, T1 shows hypointense, T2 and Flair show hyperintense and isointense signals, and some patients show fuzzy ripple-like changes with cystic changes. This is consistent with the MRI findings of the local hospital in our patient. Three years later, the MRI in our hospital showed an enlarged lesion with edema around the nodule and no cystic changes, which could be the reason for tissue repair after radiotherapy. Histological manifestations include nodules of varying sizes, scattered neuronal-like cells within the nodules, immature or blurred neuronal-like cells, circular and vacuolar nuclei, and eosinophilic cytoplasm. The vacuolar changes may be located in the cytoplasm or around the cell. Tumor cells are positive for SOX10, OLIG2, and HuC/HuD neuron-related markers, but NeuN is negative, indicating that these neuron-like cells may be immature neurons (8–10). Synaptophysin is expressed in the background glia of tumors but appears with light staining within the lesion nodules. However, synaptophysin can sometimes be seen clearly delineating the tumor nodules (11, 12). CD34 positivity indicates multipolar cells within the lesion, showing patchy positivity (13). The Ki-67 proliferation index is relatively low, indicating inert biological behavior (14). MVNT is accompanied by mutations in genes related to the MAPK pathway, BRAF genes, or FGFR genes (1). Gene mutations are mainly small indel and hotspot mutations (15, 16). Regarding treatment, MVNT can be left untreated if asymptomatic (17). Surgical resection can be chosen if the lesion is related to symptoms such as seizures and headaches (18).

MVNT has unique imaging manifestations and histological changes, and diagnosis is not difficult. But it still requires a differential diagnosis from dysplastic neuroepithelial tumor (DNT) and focal cortical dysplasia (FCD). In DNT, imaging can present as multiple nodules and is more common in children or adolescents, which often involves the superficial layer of the cortex. In addition, the morphology can also appear as multiple nodules, but within the nodules are cytoplasmic vacuolar oligodendrocytes with mucinous degeneration. The typical manifestation includes mature neurons floating within a mucinous lake; immunohistochemical staining shows NeuN positive neurons (1, 17, 19). FCD presents typical imaging changes, including subcortical abnormal signals extending from the cortex to the lateral ventricle that gradually become thinner, with a triangular tip pointing towards the ventricle or simply manifestations of cortical or subcortical high-signal shadows. Furthermore, it is worth noting that FCD is a common cause of chronic refractory epilepsy, and hypoxia caused by epilepsy causes widespread vacuolar degeneration around neurons, which is easily confused with MVNT (20). The arrangement of FCD neurons is disordered, and NeuN staining is positive, which can easily be distinguished (9).

## Conclusion

MVNT is a benign disease that does not require resection treatment. It can be diagnosed directly if the imaging morphology is typical, but follow-up is necessary to avoid misdiagnosis. Pathology also has typical manifestations: nodules of varying sizes are detected in the cortex, with vacuolar neuron-like cells visible inside and around the cells and negative immunohistochemical NeuN neuron-like cells. Some cases are accompanied by mutations in the BRAF, MAP1K1, and MAP2k1 genes. MVNT has unique imaging, pathological, and molecular characteristics, and accurate recognition of these characteristics helps to diagnose this type of neuroepithelial tumor accurately and favors a good prognosis, avoiding misdiagnosis or overtreatment.

## Data availability statement

The datasets presented in this article are not readily available because of ethical and privacy restrictions. Requests to access the datasets should be directed to the corresponding author.

## Ethics statement

The studies involving humans were approved by Shandong Provincial Hospital’s Ethics Committee for Biomedical Research Involving Human Beings. The studies were conducted in accordance with the local legislation and institutional requirements. The participants provided their written informed consent to participate in this study. Written informed consent was obtained from the participant/patient(s) for the publication of this case report.

## Author contributions

ZW: Writing – review & editing. JWM: Writing – original draft.
