# Supplementary material for: Case report: Rare case of multinodular and vacuolar neuronal tumors in the cerebellum
Source: Front Neurol. 2024 Jan 5;14:1309209. doi: 10.3389/fneur.2023.1309209 (PMC10797035; doi:10.3389/fneur.2023.1309209)
Supplement: Supplementary file 1 [file Table_1.DOCX]

Table S1 Review of literature on basic clinical information of MVNT

| Case | | Gender | | Age(years) | | Volume | | Symptom | | Location | | Treatment | | Follow up （month) | | PMID | |
| --- | --- | --- | --- | --- | --- | --- | --- | --- | --- | --- | --- | --- | --- | --- | --- | --- | --- |
| 1 | M | | 62 | | 1-5mm  （1-16） | | Vertigo | | R Parietal | | Operation | | N/A | | 31229580 | |  |
| 2 | F | | 53 | |  | | Seizure | | R Parietal | | Operation | | N/A | |  | |  |
| 3 | F | | 21 | |  | | Migraine | | L Parietal | | Operation | | 17 | |  | |  |
| 4 | M | | 23 | |  | | Seizure | | L Frontal | | Operation | | 90 | |  | |  |
| 5 | F | | 65 | |  | | Seizure | | R Parietal | | Operation | | N/A | |  | |  |
| 6 | F | | 27 | |  | | Headache | | L Frontal | | Operation | | N/A | |  | |  |
| 7 | F | | 16 | |  | | Seizure | | L Occipital | | Operation | | N/A | |  | |  |
| 8 | F | | 71 | |  | | Paresthesia | | L Frontal | | Operation | | 117 | |  | |  |
| 9 | M | | 30 | |  | | Hypoesthesia | | L Frontal | | Operation | | N/A | |  | |  |
| 10 | M | | 34 | |  | | Migraine | | R Frontal | | Operation | | 5 | |  | |  |
| 11 | F | | 46 | |  | | Seizure | | L Frontal | | Operation | | N/A | |  | |  |
| 12 | F | | 77 | |  | | Cognitive impairment | | L Frontal | | Operation | | N/A | |  | |  |
| 13 | F | | 37 | |  | | Headache | | L Parietal | | Operation | | N/A | |  | |  |
| 14 | M | | 44 | |  | | Headache | | L Frontal | | Operation | | 13 | |  | |  |
| 15 | F | | 30 | |  | | Hyperprolactinemia | | R Temporal | | Operation | | 13 | |  | |  |
| 16 | F | | 35 | |  | | Multiple sclerosis | | R Parietal | | Operation | | 111 | |  | |  |
| 17 | F | | 25 | | N/A | | Occasional Headaches | | R Parietal | | No treatment | | 12 | | 35106217 | |  |
| 18 | F | | 59 | | N/A | | Seizure | | L Temporal | | Operation | | 108 | | 28833756 | |  |
| 19 | F | | 32 | | N/A | | Seizure | | R Temporal | | Operation | | 72 | |  | |  |
| 20 | F | | 27 | | N/A | | Seizure | | L Temporal | | Operation | | 168 | |  | |  |
| 21 | M | | 62 | | N/A | | Breathing difficulties | | R Occipital | | Operation | | N/A | |  | |  |
| 22 | F | | 67 | | N/A | | Visual symptoms/ Seizure | | R Temporal | | Operation | | 24 | |  | |  |
| 23 | F | | 48 | | N/A | | Seizure/Speech arrest | | L Temporal | | Operation | | 24 | |  | |  |
| 24 | F | | 6 | | N/A | | Seizures | | L temporal | | Operation | | 17 | |  | |  |
| 25 | M | | 54 | | N/A | | Seizures | | R temporal | | Operation | | 10 | |  | |  |
| 26 | F | | 41 | | N/A | | Epilepsy | | R temporal | | Operation | | N/A | |  | |  |
| 27 | M | | 55 | | N/A | | Epilepsy | | Temporal lobe and temporal pole | | Operation | | N/A | |  | |  |
| 28 | 1/4 M 3/4 F | | 40-52(mean 45) | | 38×28mm | | Vertigo(1/4)/Headaches(3/4) | | R parietal lobe | | No treatment | | 36 | | 34169991 | |  |
| 29 |  | |  | | 27×18mm | |  | | L parietal lobe | | No treatment | | 60 | |  | |  |
| 30 |  | |  | | 54×28mm | |  | | R parahippocampal | | No treatment | | 12 | |  | |  |
| 31 |  | |  | | 25×26 mm | |  | | L parietal lobe | | No treatment | | 12 | |  | |  |
| 32 | N/A | | 54 | | 15×15mm | | Seizures | | L cerebellar | | No treatment | | 12 | | 36247696 | |  |
| 33 | M | | 52 | | 50×10mm | | Seizures | | Middel and R temporal | | Operation | | 12 | | 29111096 | |  |
| 34 | M | | 60 | | 25×17mm | | No Complaint | | L superior front | | Operation | | N/A | | 29629230 | |  |
| 35 | M | | 29 | | 27×26mm | | Headaches | | R anterior temporal lobe | | Biopsy | | N/A | | 28505008 | |  |
| 36 | M | | 10 | | N/A | | Headache | | L temporal | | Operation | | N/A | | 31950248 | |  |
| 37 | F | | 32 | | N/A | | Seizures | | L occipital | | Operation | | 15 | | 35048219 | |  |
| 38-40 | N/A | | 23-39 | | 1-3mm | | Headaches（2case) Vertigo(1case) | | cerebellar hemisphere | | No treatment | | 12 | | 31753835 | |  |
| 41 | M | | 41 | | N/A | | Seizures | | R amygdala and hippocampus | | Operation | | N/A | | 26644357 | |  |
| 42 | M | | 37 | | 26×17×14mm | | Seizure | | L parietal lobe | | Operation | | 18 | | 25146549 | |  |
| 43 | M | | 5 | | N/A | | Cerebral palsy, Spastic gait, and Toe walking. | | R hippocampus | | Operation | | N/A | | 32140033 | |  |
| 44 | F | | 34 | | N/A | | Tingling sensation in the bilateral upper and lower | | R parieto-occipital lobe | | Operation | | N/A | | 29241217 | |  |
| 45 | F | | 45 | | 7800mm^3^ | | Seizures | | L post-rolandic gyru | | Biopsy | | N/A | | 32632711 | |  |
| 46 | M | | 22 | | N/A | | Seizures | | L temporal | | N/A | | 6 | | 30108675 | |  |
| 47 | M | | 41 | | N/A | | Headache | | R medullary body | | Operation | | 24 | |  | |  |
| 48 | M | | 44 | | N/A | | Vertigo | | Vermis and L superior paravermian part | | Phenytoin treat seizures | | 6 | | 31917314 | |  |
| 49 | F | | 31 | | N/A | | Epilepsy | | L temporal | | N/A | | 36 | | 31886439 | |  |
| 50 | F | | 27 | | 40×35×15mm | | Headache | | Precuneus | | N/A | | 168 | |  | |  |
| 51 | M | | 21 | | 20×20×15mm | | Epilepsy | | R middle frontal gyrus | | Operation,antiepileptic drugs | | N/A | | 29243173 | |  |
| 52 | M | | 19 | | N/A | | Headache | | L occipital | | No treatment | | 36 | | 29405953 | |  |
| 53 | M | | 10 | | 30mm | | Headache | | L temporoparietal subcortical | | No treatment | | 24 | | 31201671 | |  |
| 54 | F | | 54 | | N/A | | Headache | | R.temporal lobe | | No treatment | | N/A | | 29428973 | |  |
| 55 | F | | 11 | | N/A | | Seizures | | L temporal | | No treatment | | 17 | |  | |  |
| 56 | F | | 19 | | N/A | | Seizures | | R.temporal lobe | | N/A | | N/A | |  | |  |
| 57 | M | | 12 | | N/A | | Seizures | | R.temporal lobe | | N/A | | N/A | |  | |  |
| 58 | M | | 38 | | 41×22mm | | Dizziness | | R.temporal | | N/A | | N/A | | 23324039 | |  |
| 59 | F | | 54 | | 17×18mm | | Dizziness | | L amygdala | | N/A | | N/A | |  | |  |
| 60 | F | | 38 | | 30×24mm | | Seizure | | R parietal | | Operation | | 8 | |  | |  |
| 61 | M | | 35 | | 36×26mm | | Episodic confusion | | R temporal | | Operation/ radiotherapy | | 72 | |  | |  |
| 62 | M | | 54 | | 26×17mm | | Seizures | | R temporal | | Operation | | 16 | |  | |  |
| 63 | F | | 31 | | 31×25mm | | Seizures | | L temporal | | Operation | | 6 | |  | |  |
| 64 | M | | 41 | | 20mm | | Confusion | | R Temporal | | Operation | | 11 | |  | |  |
| 65 | F | | 63 | | 22mm | | Numbness and tingling | | R Temporal | | Operation | | 12 | |  | |  |
| 66 | M | | 64 | | N/A | | Staring and numbling | | L temporal | | Operation | | 60 | |  | |  |
| 67 | F | | 52 | | 25×20mm | | Episodic vertigo | | L frontal | | Operation | | 36 | |  | |  |
| 68-78 | 5M /6 F | | 22-70 (37±13) | | N/A | | 4/11Headache, 2/11Meningioma screening, 1/11Bilateral upper arm praesthesia, 1/11Hearing loss, 1/11Tinitus, 1/11Dizziness, 1/11Aneurysm screening | | 7/11vermis,1/11 left cerebellar hemisphere,1/11right cerebellar hemisphere,1/11 left cerebellar peduncle | | N/A | | median 36 | | 31558497 | |  |
| 79-111 | 19F/14M | | 8-63 (mean 39, median 41) | | 7-57mm | | 16 Headache, Suspected seizure 8, 9 for other reasons | | 9/33 parietal lobe;8 frontal lobe 6 temporal lobe,2 located in the occipital lobe,8 more than one lobe | | 4/33 Operation，other no treatment | | 24-144 months (  mean 36.8 months) | | 28705817 | |  |

Abbreviations: F: Female; M: Male; R: Right; L: Left; N/A: not available
