# Supplementary material for: Case report: Rare case of multinodular and vacuolar neuronal tumors in the cerebellum
Source: Front Neurol. 2024 Jan 5;14:1309209. doi: 10.3389/fneur.2023.1309209 (PMC10797035; doi:10.3389/fneur.2023.1309209)
Supplement: Supplementary file 2 [file Table_2.DOCX]

Table S2 Review of MVNT imaging information

| Case | T1-WI | T2WI | FLAIR | Cho/Cr | Cho/NAA |
| --- | --- | --- | --- | --- | --- |
| 1-16 | isointense or hypointense | hyperintense | hyperintense | N/A | N/A |
| 17 | N/A | N/A | hyperintense | N/A | N/A |
| 18-27 | isointense or hypointense | hyperintense | N/A | N/A | N/A |
| 28 | iso-hypointense | hyperintense | hyperintense | slight increase  in Cho peak (Cho/Cr1.33) | slight drease in NAA peak (Cho/NAA 0.7) |
| 29 | iso-hypointense | hyperintense | hyperintense | increase in  Cho peak (Cho/Cr 1.18) | drease in NAA peak (Cho/NAA 1.01） |
| 30 | iso-hypointense | hyperintense | hyperintense | increase in  Cho peak (Cho/Cr 1.74) | drease in NAA peak (Cho/NAA 2.00） |
| 31 | iso-hypointense | hyperintense | hyperintense | minimal increase  in Cho peak (Cho/cr 1.03) | drease in NAA peak (Cho/NAA 0.65) |
| 32 | hypointense | hyperintense | N/A | N/A | N/A |
| 33 | N/A | hyperintense | hyperintense | N/A | N/A |
| 34 | hypointense | hyperintense | hyperintense | N/A | N/A |
| 35 | mild hyperintensity | hyperintense | hyperintense | N/A | N/A |
| 36 | N/A | hyperintense | hyperintense | N/A | N/A |
| 37 | N/A | hyperintense | N/A | N/A | N/A |
| 38-40 | hypointense | hyperintense | no suppression | N/A | N/A |
| 41 | hypointense | hyperintense | hyperintense | N/A | N/A |
| 42 | iso-intense | hyperintense | hyperintense | N/A | N/A |
| 43 | hypointense | hyperintense | hyperintense | N/A | N/A |
| 44 | N/A | hyperintense | hyperintense | N/A | N/A |
| 45 | isointense | hyperintense | hyperintense | 1.49 | 1.54 |
| 46 | hypointense | hyperintense | hyperintense | 1.36 | 0.77 |
| 47 | hypointense | hyperintense | partial suppression | 0.75 | 0.72 |
| 48 | hypointense | hyperintense | partial suppression | 0.99 | 0.69 |
| 49 | N/A | hyperintense | hyperintense | N/A | N/A |
| 50-51 | hypointense | hyperintense | hyperintense | N/A | N/A |
| 52 | N/A | hyperintense | hyperintense | N/A | N/A |
| 53 | hypo/iso-intense | hyperintense | N/A | N/A | N/A |
| 54 | N/A | hyperintense | N/A | N/A | N/A |
| 55-57 | N/A | hyperintense | N/A | N/A | N/A |
| 58 | N/A | hyperintense | hyperintense | N/A | N/A |
| 59 | N/A | hyperintense | hyperintense | N/A | N/A |
| 60 | hypointense | hyperintense | hyperintense | N/A | N/A |
| 61 | N/A | hyperintense | hyperintense | N/A | N/A |
| 62 | isointense | hyperintense | hyperintense | N/A | N/A |
| 63 | hypointense | hyperintense | hyperintense | N/A | N/A |
| 64 | N/A | hyperintense | N/A | N/A | N/A |
| 65 | hypointense | hyperintense | hyperintense | N/A | N/A |
| 66 | isointense | hyperintense | hyperintense | N/A | N/A |
| 67 | N/A | hyperintense | hyperintense | N/A | N/A |
| 68-78 | 12/13hypointense | all hyperintense | all hyperintense | N/A | N/A |
| 79-111 | isointense | hyperintense | N/A | N/A | N/A |

Abbreviations: N/A: not available
