# Supplementary material for: Case report: Rare case of multinodular and vacuolar neuronal tumors in the cerebellum
Source: Front Neurol. 2024 Jan 5;14:1309209. doi: 10.3389/fneur.2023.1309209 (PMC10797035; doi:10.3389/fneur.2023.1309209)
Supplement: Supplementary file 3 [file Table_3.DOCX]

Table S3 Review of pathological and molecular genetic information in MVNT

| Case | IHC positive | IHC negative | Gene |
| --- | --- | --- | --- |
| 18-27 | SOX2, CD34, MCM2 | PAX6, NPY | SUFU,E2H2,DEPDCS,NLRL3,CIC,PIK3CA,SMO,TP53 |
| 33 | PGP9.5,ATRX,OLIG2,SOX10,P16,Nestin, Syn | NeuN,GFAP,CgA, CD34, IDH1, P53 | N/A |
| 34 | HuC/HuD,NeuN,Syn,Olig-2,ki67<1% | P53,CD34,IDH1 | N/A |
| 35 | Syn, ki67<1%, | IDH1,P53,BRAF,CgA | No somatic mutations within the hotspot regions of 50 genes |
| 36 | Syn,,Olig2,GFAP,,KI67 1% | NeuN, IDH1, BRAF |  |
| 37 | Olig2 | NeuN,GFAP,MAP2,  CD34,P53,IDH1,PHH3 | MAPK2K1 p.Q56_V60del |
| 41 | HuC/Hud,Olig2,P62,Syn,MAP-2,GFAP,S-100,INA,vimtin, CD34, Ki-67 | NeuN, Nestin, IDH1 | N/A |
| 42 | HuC/HuD,Olig2,Syn,  CD34+, Ki67<1% | GFAP, NeuN, CgA, P53 N/A  BFAFV600E, IDH1 | |
| 43 | Olig2, NF, ki67<2% | NeuN | N/A |
| 45 | GFAP,Syn,Olig2, ATRX, Ki67 2%. | NeuN,CD34,IDH1/2,BRAF(VE1) | N/A |
| 54 | p-ERK | N/A | BRAF.Pl597r |
| 55 | N/A | N/A | BRAF p.G469S |
| 56 | N/A | N/A | MAP2K1 P.K57_E62delinsK |
| 57 | N/A | N/A | FGFR-INA gene fusion |
| 62 | N/A | N/A | MEK1(MAP1K1)p.Q56P(c.167A>C) |
| 67 | CD34,Olig2,Syn,HUC/HUD | GFAP, NenN | N/A |
| 79-111 | CD34,ATRX,P53,ki67 | IDH1, GFAP | N/A |

Abbreviations: N/A: not available
